# Decreased expression of Sushi Domain Containing 2 correlates to progressive features in patients with hepatocellular carcinoma

**DOI:** 10.1186/s12935-016-0286-5

**Published:** 2016-02-29

**Authors:** Xin-rui Liu, Cui-xia Cai, Li-min Luo, Wen-Ling Zheng, Rong Shi, Jun Zeng, You-qin Xu, Min Wei, Wen-li Ma

**Affiliations:** Institute of Genetic Engineering, School of Basic Medical Sciences, Southern Medical University, No.1838, Baiyun Road North, Guangzhou, China; Centre for Liver Disease, 458th Hospital of PLA, Guangzhou, 510602 China

**Keywords:** SUSD2, Hepatocellular carcinoma, Tissue microarray, Immunohistochemistry, Cell proliferation, Invasion, Migration, Apoptosis

## Abstract

**Background:**

Sushi Domain Containing 2 (SUSD2) has been identified as a regulator of colon and breast cancer. Increasing evidence suggests that SUSD2 plays a key role in tumorigenesis. However, the SUSD2 expression status and its functions in hepatocellular carcinoma (HCC) are still unrevealed. In the present study, we intended to investigate SUSD2 expression status and its correlation with the clinicopathological features in HCC patients. Furthermore,we examined the influence of SUSD2 on the proliferation, apoptosis, invasion and migration of the HCC cell lines HepG2 and SMMC7721.

**Methods:**

We evaluated the SUSD2 expression in HCC tissues and paired normal liver tissues by quantitative real-time PCR and western blotting analysis. The clinicopathological significance of SUSD2 was investigated by immunohistochemistry (IHC) on a HCC tissue microarray. Receiver operating characteristic (ROC) analysis was applied to determine the optimal cut-off score for positive expression of SUSD2. The correlation between SUSD2 protein expression and clinicopathological features of HCC was analyzed by Chi square test. The cell proliferation, apoptosis, invasion and migration potential were observed to detect the functions of SUSD2 in HCC cells.

**Results:**

Decreased expression of SUSD2 mRNA and protein were observed in the majority of HCC tissues, compared with paired normal liver tissues. When SUSD2 high expression percentage was determined to be above 52.5 % (area under ROC curve = 0.769, *P* = 0.000), low expression of SUSD2 was observed in 62.2 % (112/180) of HCC tissues and high expression of SUSD2 was observed in all normal liver tissues (16/16) by IHC. Decreased expression of SUSD2 in patients was correlated with high histological grade (χ^2^ = 5.198, *P* = 0.023), advanced clinical stage (χ^2^ = 30.244, *P* = 0.000), pT status (χ^2^ = 33.175, *P* = 0.000), pN status (χ^2^ = 4.785, *P* = 0.029), pM status (χ^2^ = 4.620, *P* = 0.032). Down-regulation of SUSD2 promoted cell proliferation,invasion and migration,reduced the cell apoptosis.

**Conclusions:**

Our findings suggest that SUSD2 may play as a tumor suppressor in HCC cells and could be served as an additional potential marker for diagnosis.

## Background

Hepatocellular carcinoma (HCC) is the sixth most common malignancy worldwide. Population-based studies show that the incidence rate continues to approximate the death rate, indicating that most of the patients who develop HCC die of it [1]. Surgical resection or liver transplantation is usually considered curative treatment for early HCC [2]. Unfortunately, exceeding 50–70 % of HCC patients present with advanced disease that is not amenable to surgical resection or transplantation and thus the 5-year relative survival rate for patients with HCC is only 7 % [3]. HCC always develops in the setting of liver cirrhosis associated with hepatic regeneration after tissue damage caused by a chronic hepatitis B or C virus infection, chronic alcohol consumption or metabolic influences [4]. The mutations occurring in single or multiple oncogenes or tumor suppressor genes are increasingly recognized as critical determinants of carcinogenesis [5]. Molecular analysis of HCC revealed genetic and epigenetic alterations that lead to the deregulation of key proto-oncogenes and tumor suppressor genes including P53, β-catenin, ErbB receptor factor, p16, E-cadherin, and cyclooxygenase 2 in this cancer [6, 7]. However, due to lack of early diagnosis methods and traditional stratification schemes based on clinical characteristics such as AJCC TNM stage and BCLC stage provide limited prognostic guidance in the management of HCC patients, the majority of HCC cases were diagnosed at terminal stages and the long-term prognosis remained unfavorable [8, 9]. Biomarkers that can predict clinical outcome of specific cancers are helpful for the development of therapeutic strategies in the earlier stages of the diseases [10]. Thus, it is prevalent to discovery novel biomarkers for accurate diagnosis, prognosis and individualized medication of HCC.

SUSD2 (Sushi Domain Containing 2) was first identified in mouse as a tumor-reversing gene. Sugahara T reported that the mouse homolog SUSD2 was downregulated in Ki3T3 cells compared with NIH3T3 cells and indicated that upregulation of SUSD2 in HT1080 cells and HeLa cells inhibits clonogenicity, anchorage-independent growth, migration, and invasion [11, 12]. Their work indicated a possible tumor suppressive role of mouse SUSD2. Recently, the human SUSD2 has been shown is located on chromosome 22 and encodes an 822-amino acid type I membrane protein containing somatomedin B, AMOP, von Willebrand factor type D, and Sushi domains, which are frequently found in molecules playing important roles in cell–cell and cell–matrix adhesion [13]. Currently, SUSD2 has been reported to be dysregulated in some cancers and accumulating evidences suggested that reduced expression of SUSD2 plays a key role in tumorigenesis. Pan et al. showed that SUSD2 expression was downregulated both on mRNA and protein levels in most of colon cancer and cell lines. SUSD2 is indispensable for the growth inhibitory effect of CSBF/C10orf99 on colon cancer cells and recombinants SUSD2-Fc can block its function [14]. However, Watson et al. reporteded that SUSD2 was high expression in human breast cancer. Their study described that the interaction of SUSD2 with Gal1 increased the invasion of breast cancer cells and contributed to a potential immune evasion mechanism through induction of apoptosis of Jurkat T cells [13]. Their results suggested SUSD2 as an oncogene in breast cancer. Up to present, the published work suggests that the status of SUSD2 expression may be an important factor in tumorigenesis, but the function in different cancers may be different; furthermore, little is known about the effect of SUSD2 expression on HCC patients and prognosis.

In this study, we examined the expression pattern of SUSD2 in HCC patients, and the correlation between its expression levels with clinicopathological variables of HCC. We observed a significant low expression level of SUSD2 in patients with HCC. Reduced expression of SUSD2 was correlated to progressive features in HCC patients. Furthermore, we have investigated the mechanism of SUSD2 in HCC cell lines. These results show that exogenous overexpression of SUSD2 suppresses the cell proliferation, invasion and migration of HCC cells and promotes the cell apoptosis in vitro.

## Methods

### Patients and tissue specimens

In this study, 8 pairs of fresh HCC tissues and adjacent nonmalignant liver tissue were collected from patients for Western blotting and qRT-PCR analysis between 2012 and 2014. A total of 180 paraffin-embedded tissues diagnosed with HCC at the Centre for Liver Disease, 458th Hospital of PLA (Guangzhou, China), between January 2002 and December 2013 was retrieved for TMA construction and IHC analysis. Of the 180 HCC samples,16 matched adjacent non-malignant tissues were available as controls. None of the patients had received preoperative anticancer treatment. All patients in this study were classified according to the 2002 TNM (tumor-node-metastasis) staging of International Union Against Cancer and the American Joint Committee on Cancer (AJCC). The median age of patients was 42 years (range, 16–98 years), and 135 (75.0 %) were males, 45 (30.0 %) were females, clinicopathological features of patients including age at diagnosis, sex, histological grade, clinical stage and pTNM status. Written informed consent was obtained from all patients for use of the tissue samples and clinical records. The study protocol was performed under the approval by the Ethic Committee of the 458th Hospital of PLA.

### Western blot analysis

Total protein was isolated from 8 pairs of fresh HCC tissue and adjacent nonmalignant liver tissue. Equal amount of tissue lysates were resolved by SDS-polyacrylamide gel electrophoresis (PAGE) and electrotransferred on a polyvinylidene difluoride membrane (Millipore). The membranes were blocked in 5 % non-fat dry milk diluted with TBST (10 mM Tris–HCl and 0.05 % Tween 20) at 4 °C overnight. The membranes were then incubated with primary antibodies at room temperature for 2 h, followed by incubation with appropriate secondary antibodies(1:1000, Santa Cruz Biotechnology, USA) at room temperature for 2 h. The primary antibodies were polyclonal rabbit anti-human antibody against SUSD2 (1:500 dilution; Sigma) and monoclonal rabbit anti human antibody against β-actin (1:2000 dilution; Cell Signaling Technology). The membranes were washed with TBST for three times, and the immunoreactive band were visualized using the ECL plus Western blot detection kit.

### Quantitative reverse transcription PCR (qRT–PCR)

Total RNA was extracted from eight pairs of fresh HCC tissue and adjacent nonmalignant liver tissue using RNAiso Plus reagent (Takara, Dalian, China). Qualified total RNAs were reversely transcribed into first-strand cDNAs by using the PrimeScript^®^ RT reagent Kit (Takara, Dalian, China). For the *SUSD2* gene, the forward primer was 5′-CTCCAATGACTGCCGCAACTA-3′, and the reverse primer was 5′-GAACATCCTTTCAGGTCCATCC-3′. For the β-actin gene,the forward primer was 5′-CTCCAATGACTGCCGCAACTA-3, and the reverse primer was 5′-GAACATTCCTTTCAGGTCCATCC-3′. Realtime PCR was carried out using an ABI 7500 real-time PCR amplifier (Applied Biosystems, USA) to determine the expression pattern of SUSD2 mRNA in each of the HCC sample as well as the paired adjacent non-cancerous tissue. Quantitative real-time PCR was performed by using the SYBR^®^ Premix Ex TaqTM II Kit (Takara, China)in a total volume of 20 μl. The PCR conditions were as follows: 95 °C for 30 s, 40 cycles of amplification at 95 °C for 5 s and 60 °C for 34 s, followed by additional dissociation stage for testing reaction specificitywas performed to generate a melting curve for confirmation of amplification specificity. β-actin was used as the reference gene. The relative levels of gene expression were represented as ΔCt = Ct_(gene)_−Ct_(reference)_, and the fold change of gene expression was calculated by the 2^−ΔΔCt^ method. Experiments were repeated in triplicate.

### Construction of tissue microarrays (TMA) And immunohistochemistry

Tissue microarray was constructed in accordance with previously described methods [15]. In brief, the slides were reviewed by a pathologist to determine and mark out representative tumor areas. Duplicates of 0.6-mm diameter cylinders were punched from representative areas of individual donor tissue block, and then re-embedded into a recipient paraffin block in a defined position, using a tissue arraying instrument (Beecher Instruments, Silver Spring, MD). In our constructed liver tissue-TMA, three cores of a sample were selected from each primary HCC and normal liver tissue. Then, for the need of immunohistochemistry analysis, TMA block would be cut into several 5-μm sections. The TMA block contained 180 HCC and 16 specimens of normal liver tissues. For immunohistochemistry, the TMA slides were dried overnight at 37 °C, deparaffinized, rehydrated. Endogenous peroxidase activity was blocked with 0.3 % hydrogen peroxide for 20 min. For antigen retrieval, slides were heated in a microwave oven for 10 min in 10 mmol/L citrate buffer, pH 6.0. After blocking with 5 % normal goat serum at room temperature for 30 min, the slides were then incubated with SUSD2 primary antibody (Rabbit polyclonal, Sigma) at a dilution of 1:100 at 4 °C overnight and subsequently incubated with polymer peroxidase-labeled secondary antibody (Zhongshan biotech, China) at a concentration of 1:100 for 30 min at 37 °C. The final detection was visualized by using DAB Horseradish Peroxidase Color Development Kit (Beyotime, China) after hematoxylin counterstaining. The phosphate-buffered saline was set as negative control.

### Evaluation of immunohistochemistry results and selection of cut-off scores

Immunoreactivity for SUSD2 protein was scored by semi-quantitative method by evaluating the number of positive tumor cells over the total number of tumor cells. Scores were assigned by using 5 % increments (0, 5, 10… 100 %). SUSD2 expression was assessed by three independent pathologists (Cuixia.C, R.S and J.Z) who were blinded to clinicopathologic data. Their conclusions were in complete agreement in approximately 85 % of the cases, which identified this scoring method was highly reproducible. If two or all of them were consistent with the results they reported, the value was selected. If the results were completely different, all three worked together to confirm the score.

Receiver–operator curve (ROC) analysis was applied to determine cut-off scores for tumor ‘‘high expression’’ by using the 0, 1-criterion. At the SUSD2 score, the sensitivity and specificity for each outcome under study was plotted, thus generating an ROC. The score closest to the point with both maximum sensitivity and specificity (i.e. the point [0.0, 1.0] on the curve) was selected as the cut-off score. Tumors designated as low expression of SUSD2 were those with the scores below or equal to the cut off value, while tumors of high expression were those with scores above the value. For the need of ROC curve analysis, the clinicopathologic characteristics were dichotomized: T status (T1–T2 versus T3–T4), N status (N0 versus N1), clinical stage (I–II versus III–IV), histological grade (G1–G2 versus G3).

### Cell culture

The human HCC cell lines, HepG2 and SMMC7721 were obtained from laboratory preservation. These cell lines were cultured in Dulbecco’s modified Eagle’s medium (DMEM High Glucose, Hyclone, USA) supplemented with 10 % heat-inactivated fetal bovine serum (Gibco, USA), penicillin (100 units/ml) and streptomycin (100 μg/ml) at 37 °C in a humidified 5 % CO2 atmosphere.

### Vector construction and cell transfection

For SUSD2 overexpression, the open reading frame of SUSD2 was cloned into the multiple cloning site of the pcDNA3.1 between Hind III–EcoR I restriction sites and the empty pcDNA3.1 vector was used as control. psi-mH1-SUSD2 containing a shRNA sequence (5′-GAACGAGACGCGTTGGCAATA-3′) against SUSD2 was purchased from Guangzhou FulenGen. shRNA-scramble (5′-GCTTCGCGCCGTAGTCTTA-3′) was used as control. The plasmid transfection was optimized using Lipofectamine 2000 according to the manufacturer’s instructions. Briefly, lipofectamine and plasmid were diluted separately in serum-free Opti-MEM (Gibco) and incubated at room temperature for 5 min. Afterward, the two solutions were gently mixed and incubated for 20 min. Finally, the mixture was added to plated cells, and after 2 days, the cells were analyzed using the following assays.

### Cell proliferation assays

To determine the impact of SUSD2 on cell proliferation, HepG2 cells transfected with pcDNA3.1-SUSD2 or empty pcDNA3.1 vector, SMMC7721 cells transfected with psi-mH1-SUSD2 or shRNA-scramble were seeded onto 96-well plate (Corning inc, Corning NY) at a density of 1 × 104 cells per well. Then at time points of 0, 12, 24, 36, 48 h, the cell viability rate was assessed using cell counting kit-8 (KeyGEN BioTECH, China). 10 ul CCK-8 solution was added to each well and incubated for another 1 h and a microplate reader was used to measure the absorbance of each well at 450 nm. All the experiments were independently repeated three times.

### Analysis of cell apoptosis

The transfected HCC cell lines undergoing apoptosis were distinguished from live and necrotic cells by using Annexin-V and Propidium iodide (PI) staining Kit (KeyGEN BioTECH, China). Briefly, the concentration of transfected HCC cells was set to approximately 1 × 105 cells/mL and were incubated with annexin-V/PI for 15 min at room temperature. Cells were then analyzed by means of flow cytometry using a two-color fluorescence-activated cell sorting (FACS) analysis (Beckman Coulter, cytomics FC 500, CA).

### Wound–healing Assay

The ability of migration of HCC cells were analyzed by wound-healing assays. After 48 h of transfection, 5 × 10^5^ transfected cells were seeded on 6-well plates with 10 % FBS. Then the cells were grown to 80 % confluence, wounds were created by scraping the cells with a 100-μl pipette tip. After washed with PBS three times, cells were cultured by serum-free medium during the process. The microscope was used to photograph the migrated distance of cells after 48 h.

### Invasion assays

For invasion assays, a total of 8 × 104 various cells in 100 μl serum-free DMEM medium were seeded in a Matrigelcoated chamber (8 μm pore size; BD Biosciences) and the lower chamber was immediately filled with 500 μl of DMEM medium with 10 % FBS as a chemoattractant. After 24 h of incubation in a humidifiedatmosphere containing 5 % CO2 at 37 °C, the non-invading cells are removed from the upper chamber by a cotton swab and the membranes were then fixed with methanol and stained by 0.1 % crystal violet.

### Statistical analysis

Statistical analysis was performed by using the SPSS statistical software package (standard version 13.0; SPSS, Chicago, IL, USA). Receiver–operator curve (ROC) analysis was performed to determine the cut-off scores for high expression of SUSD2. The relationship between SUSD2 protein expression and HCC patients’ clinicopathologic features was estimated by χ^2^-test. Differences between groups were analyzed using Student’s *t* test and the *P* value less than 0.05 was considered to be statistically significant.

## Results

### The expression level of SUSD2 mRNA and protein in paired HCC and adjacent normal liver tissues

**Fig. 1:**
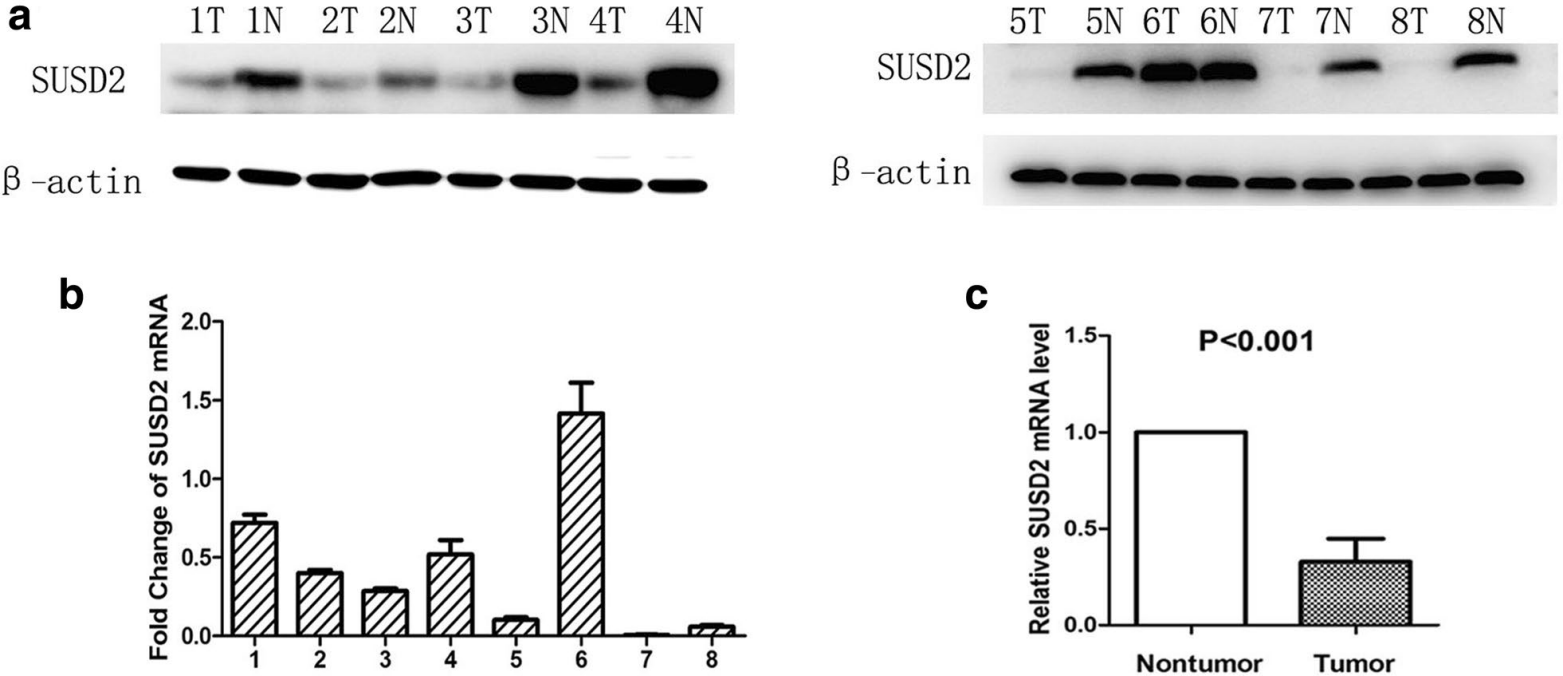
Expression of SUSD2 in HCC tissues and paired normal liver tissues detected by western blotting and qRT-PCR. **a** Low expression of SUSD2 protein was detected by Western blotting in HCC cases (7/8) compared with paired normal liver tissues. β-actin was used as internal control. T, HCC tissues; N, paired normal liver tissues. **b** Fold changes (2^−△△Ct^ values) by qRT-PCR showed a reduced expression of SUSD2 mRNA in the majority of HCC cases (7/8) compared with paired normal liver tissues. Expression levels were normalized for β-actin. **c** Significant differences of SUSD2 mRNA expression between the HCC and paired normal liver tissues (*P* < 0.05)

To assess the protein and mRNA expression of SUSD2 in HCC, western blotting and qRT-PCR was employed to measure the expression status in 8 pairs of fresh HCC tissue and adjacent normal prostate tissues. Western blotting showed that 7/8 HCCs displayed reduced level of SUSD2 compared with the adjacent normal liver tissues (Fig. 1a). Similar results for mRNA expression were observed using qRT-PCR. The results showed that in 7of the 8 sample pairs, mRNA fold changes (the 2^−△△Ct^ values) were less than 1 between HCC and adjacent normal liver tissue which indicated the SUSD2 mRNA expression was downregulated in HCC tissues compared to the adjacent normal liver tissues (Fig. 1b). The mean fold change is 0.385 and paired t test showed that difference between the two groups was statistically significant (Fig. 1c) (*P* < 0.05).

### IHC Analysis of SUSD2 expression and selection of cut-off score for high expression of SUSD2 in HCC tissues

**Fig. 2:**
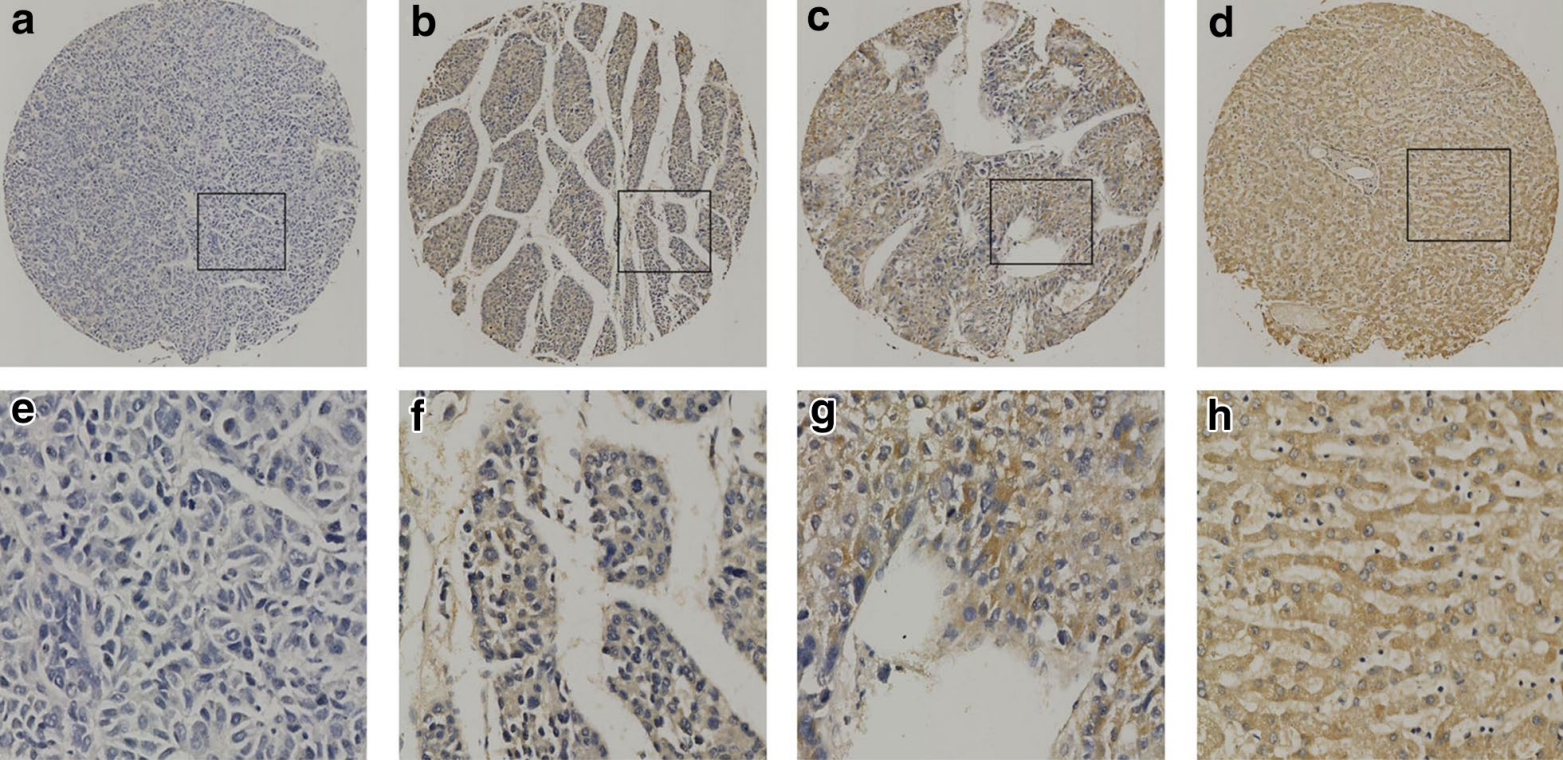
The expression of SUSD2 protein in HCC tissues and normal liver tissues by IHC on the TMA. **a** Weak staining of SUSD2 was detected in a HCC tissue. **b** Moderate staining of SUSD2 was detected in a HCC tissue, in which more than 70 % of HCC cells stain positively for SUSD2 protein in the cytoplasm. **c** Strong staining of SUSD2 was detected in a HCC case, in which more than 90 % HCC cells showed positive staining of SUSD2 protein in the cytoplasm. **d** Strong staining of SUSD2 was detected in a normal liver tissue, in which about 100 % cells showed positive staining of SUSD2 protein in the cytoplasm. **e–h** demonstrate the higher magnification (400×) from the area of *black square* in (**a–d**), respectively

**Fig. 3:**
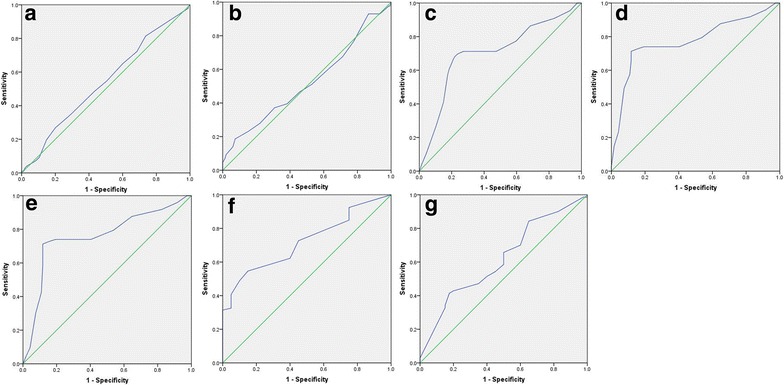
Selection of the optimum cut-off score for positive expression of SUSD2 by receiver operating characteristic (ROC) analysis. Various ROC curves were plotted by sensitivity and specificity for each clinical characteristic,including: age (**a**), sex (**b**), histological grade (**c**), clinical stage (**d**), pT status (**e**), pN status (**f**), pN status (**g**)

We further examined the expression and subcellular localization of the SUSD2 protein by IHC in a TMA, which including 180 cases of HCC and 16 cases of normal liver tissue. For SUSD2 IHC staining in HCC tissues and normal liver tissues, SUSD2 is primarily expressed in the cytoplasm within tumor cells (Fig. 2). The ROC for each clinicopathological feature (Fig. 3) show the point on the curve closest to (0.0, 1.0), which maximizes both sensitivity and specificity for the outcome. Tumors with scores above the obtained cut-off value were considered as highly expressed SUSD2, leading to the greatest number of tumors correctly classified as having or not having the clinical outcome. The corresponding area under the curve (AUC) (95 % confidence interval [CI]) are listed in Table 1. In our current study, optimal cut-off score for SUSD2 was determined by the ROC curve for clinical stage which showed the shortest distance to the point (0.0, 1.0) and could maximize both the sensitivity and specificity. According to ROC analysis, expression percentage for SUSD2 above the critical value 52.5 % was defined as positivity. The positive expression of SUSD2 was detected in 16⁄16 (100 %) of non-cancerous adjacent tissues. However, the high expression of SUSD2 was only detected in 37.8 % (68 of 180) HCC cases and the remaining 62.2 % (112 of 180) were scored as having no or low SUSD2 expression.

**Table 1:** Area under the receiver operating characteristic curve for each clinicopathological features

Feature	AUC (95 % CI)	*P* value
Age	0.540 (0.458–0.622)	0.338
Sex	0.524 (0.420–0.627)	0.636
Histological grade	0.704 (0.621–0.788)	0.000
Clinical Stage	0.769 (0.693–0.844)	0.000
pT status	0.758 (0.682–0.834)	0.000
pN status	0.721 (0.626–0.816)	0.001
pM status	0.619 (0.511–0.726)	0.039

*AUC* area under the curve, *CI* confidence interval

### Correlations between SUSD2 expression and clinicopathological parameters of HCC patients

The relationship between SUSD2 scores with respect to patient clinicopathological features was detailed in Table 2. The results demonstrated that low expression of SUSD2 was positively correlated with tumor advanced clinical stage (χ^2^ = 30.244, *P* < 0.05), pT status (χ^2^ = 33.175, *P* < 0.05), pN status (χ^2^ = 4.785, *P* < 0.05) and histological grade (χ^2^ = 5.198, *P* < 0.05), pM status (χ^2^ = 4.620, *P* < 0.05). However, there was no statistically significant correlations between SUSD2 expression and other clinicopathologic features, such as patient gender (χ^2^ = 1.093, *P* > 0.05), age at diagnosis (χ^2^ = 0.31, *P* > 0.05).

**Table 2:** Relationship between SUSD2 expression and clinicopathological characteristics of HCC patients

Characteristics	Total	SUSD2 staining		Pearson Chi square	*P *value^b^
		Negative (%)	Positive (%)		
Ages (years)				0.31	0.861
≤42^a^	45	27 (60.0)	18 (40.0)		
>42	135	79 (58.5)	56 (41.5)		
Sex				1.093	0.296
Female	45	29 (64.4)	16 (35.6)		
Male	135	75 (55.6)	60 (44.4)		
Histological grade				5.198	0.023
G1–G2	154	88 (57.1)	66 (42.9)		
G3	26	21 (80.8)	5 (19.2)		
Clinical stage				30.244	0.000
I–II	66	23 (34.8)	43 (65.2)		
III–IV	114	87 (76.3)	27 (23.7)		
pT status				33.175	0.000
T1–T2	76	24 (31.6)	52 (68.4)		
T3	104	78 (75)	26 (25)		
pN status				4.785	0.029
N0	160	63 (39.4)	97 (60.6)		
N1	20	13 (65)	7 (35)		
pM status				4.620	0.032
M0	164	67 (40.9)	97 (59.1)		
M1	16	11 (68.7)	5 (31.3)		

^a^ Median age

^b^ P value are from Chi square test

### Effects of SUSD2 on the proliferation of HCC Cells in vitro

**Fig. 4:**
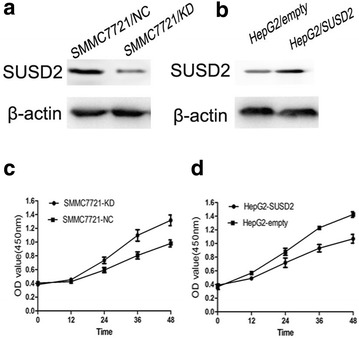
Alternations of SUSD2 expression modulated the growth of HCC cells in vitro. **a** Western blotting revealed that psi-mH1-SUSD2 transduction down-regulated SUSD2 in SMMC-7721 cells at the protein level. **b** Western blotting revealed that pcDNA3.1-SUSD2 transduction up-regulated SUSD2 in HepG2 cells at the protein level. **c** Growth curves showed that down-regulation of SUSD2 promoted the growth of SMMC-7721 cells by CCK8 assay in vitro. **d** Growth curves showed that upregulation of SUSD2 inhibited the growth of HepG2 cells by CCK8 assay in vitro

To investigate the function of SUSD2 in the tumorigenesis of HCC, we changed the expression level of SUSD2 in HepG2 cells and SMMC7721 cells. The HepG2 cells displayed a low level of SUSD2 and SMMC7721 displayed a high level of SUSD2. Then, we up-regulated SUSD2 in HepG2 cells by the transient transfection of pcDNA3.1-SUSD2 and down-regulated expression of SUSD2 in SMMC7721 cells by the transient transfection of psi-mH1-SUSD2 (Fig. 4a, b). To explore whether SUSD2 affects the growth of HCC cells, we conducted cell proliferation by using CCK-8 assays. The results showed that down-regulation of SUSD2 significantly increased the growth of SMMC7721/KD cells in comparison with control cells (Fig. 4c) *(P* < 0.05). On the contrary with the down-regulation results, the up-regulation of SUSD2 significantly inhibited the HepG2/SUSD2 proliferation in comparison with control cells (Fig. 4d) (*P* < 0.05). Taken together, the results indicate that SUSD2 functions as a tumor suppressor and play a role in cellular proliferation.

### Effects of SUSD2 on the cell apoptosis of HCC Cells

**Fig. 5:**
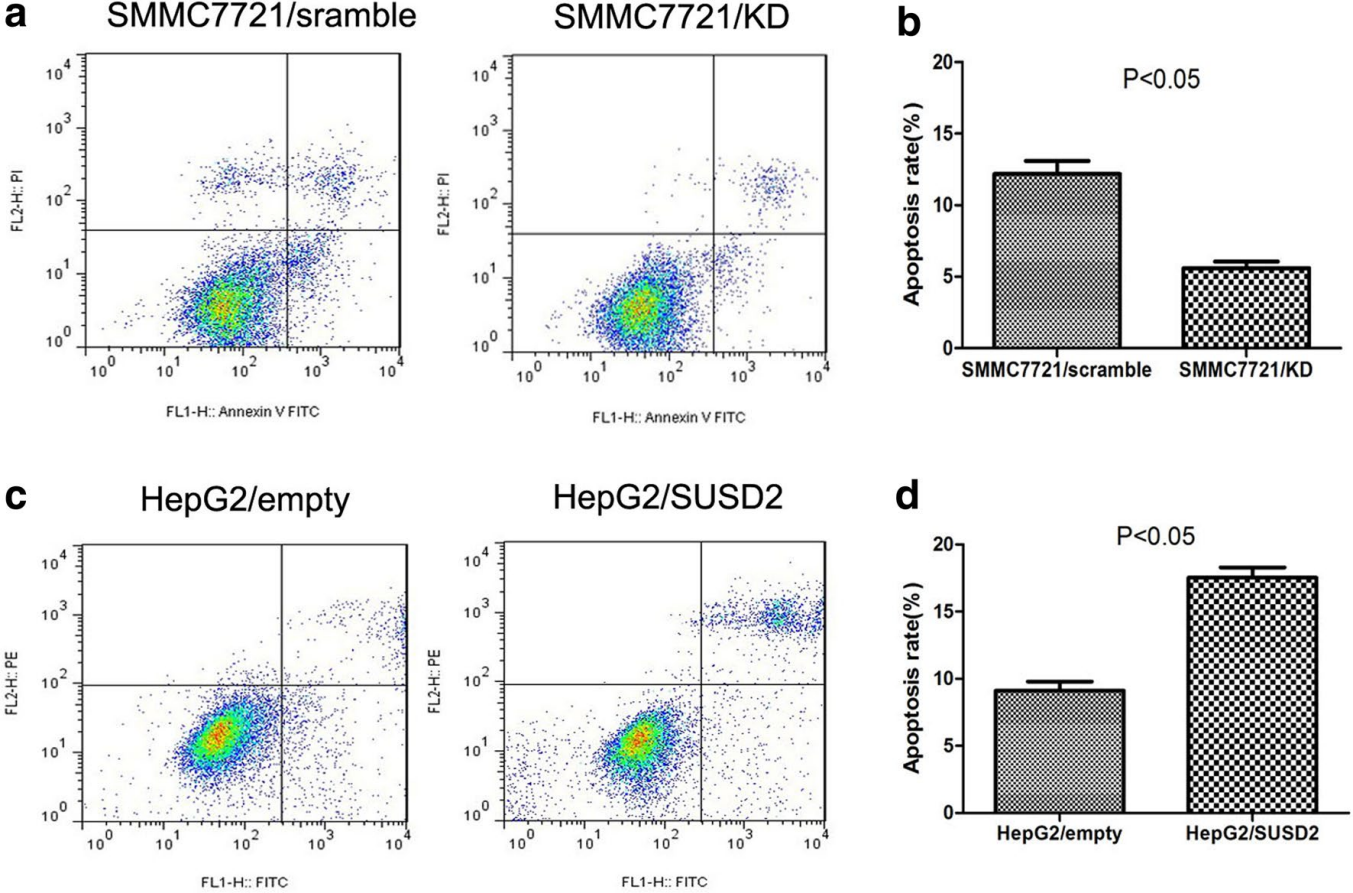
The influence of SUSD2 on apoptosis of HCC cells.Annexin-V/PI flow cytometric analysis of apoptosis in HCC cells. **a**, **b** Downregulation of SUSD2 could significantly inhibit apoptosis of SMMC-7721cells compared with the control cells (*P* < 0.05); **c**, **d** Transfection with pcDNA3.1-SUSD2 significantly promoted apoptosis of HepG2 cells compared with the control cells (*P* < 0.05)

To examine whether SUSD2 expression influences the cell apoptosis of HCC cells, we conducted cell apoptosis using Annexin V-FITC/PI double staining by flow cytometry. Annexin V-FITC+/PI- signifies the presence of apoptotic cells at an early stage and Annexin V-FITC+/PI+ signifies the late apoptotic cells. The results indicate that SUSD2 knock down significantly reduced cell apoptosis compared with cells treated with scramble shRNA (Fig. 5a, b) (*P* < 0.05). Furthermore, we carried out a similar experiment and found that HCC cells with SUSD2 overexpression promoted cell apoptosis (Fig. 5c, d) (*P* < 0.05).

### Effects of SUSD2 on the HCC cell migration and invasion

**Fig. 6:**
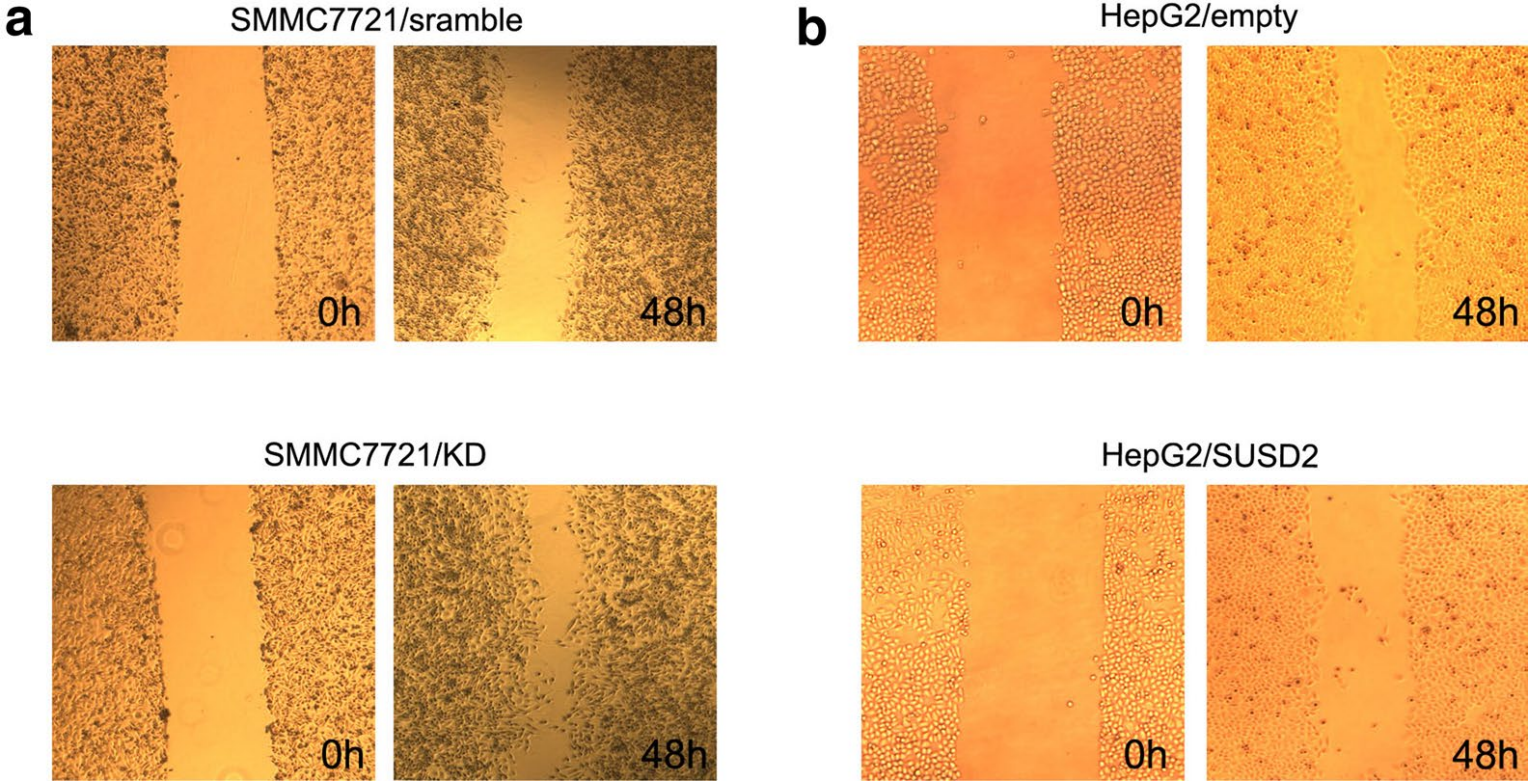
Ectopic expression of SUSD2 are responsible to migration in HCC cells. Migratory capabilities were analyzed by wound-healing assay in HCC cells. **a** Downregulation of SUSD2 could have more migratory capabilities of SMMC-7721 cells than their control groups. **b** Transfection with pcDNA3.1-SUSD2 could inhibit migratory capabilities of HepG2 cells compared with the control groups

**Fig. 7:**
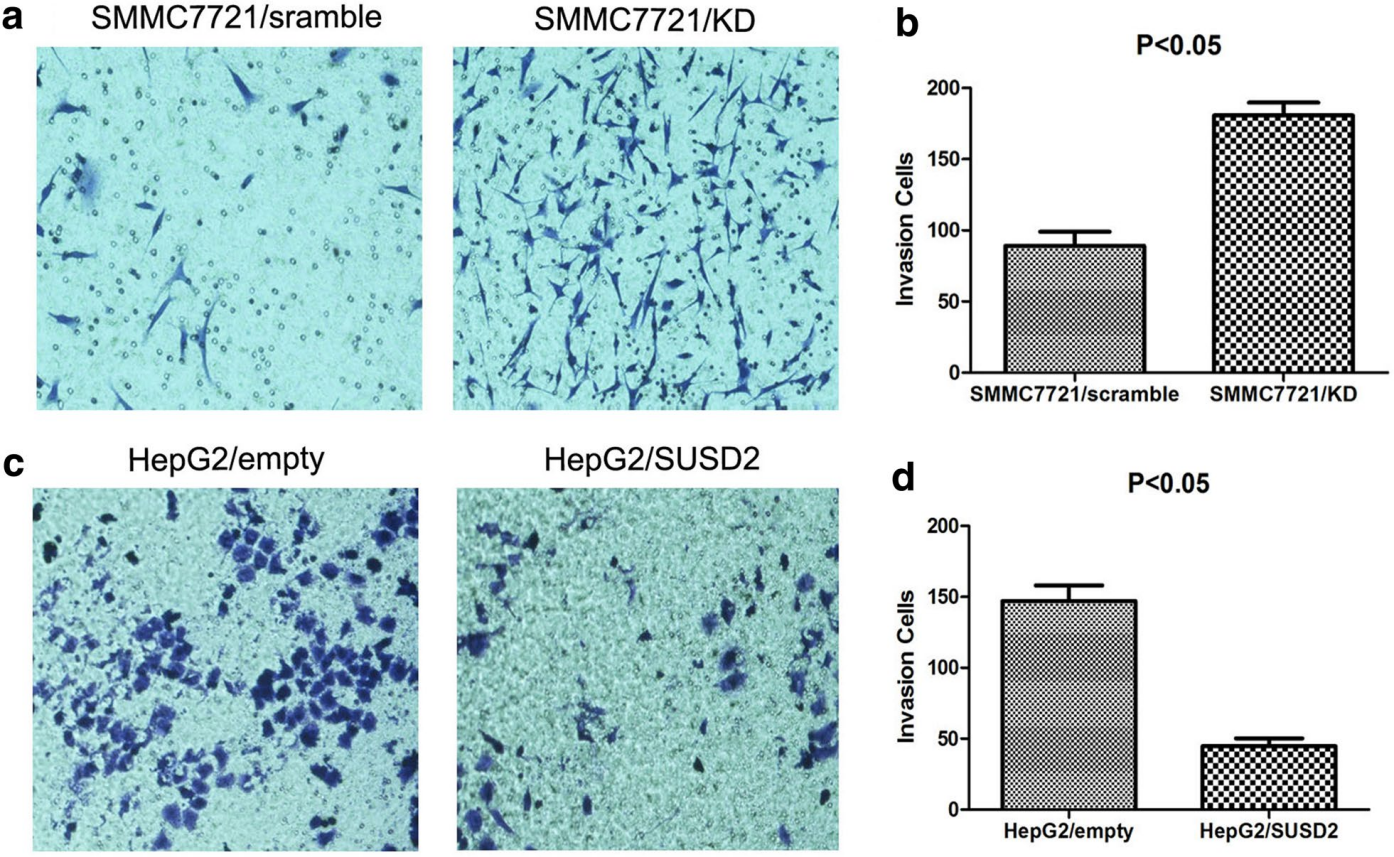
The function of SUSD2 on invasion was assessed by transwell assay. **a**, **b** Downregulation of SUSD2 increased the invasive potential of SMMC-7721 cells compared with the control groups (*P* < 0.05). **c**, **d** Overexpression of SUSD2 decreased the invasive potential of HepG2 cells compared with the control groups (*P* < 0.05)

We next conducted wound-healing assay and transwell assays to examine whether SUSD2 expression influence the ability of HCC cells on cell migration and invasion.The results showed that SUSD2 knock down significantly increase cellular migration of SMMC7721 cells (Fig. 6a). And SUSD2 up-regulation significantly decreased cellular migration of HepG2 cells (Fig. 6b). Next, we carried out an experiment to compare the ability of SUSD2 on invasion. As shown in Fig. 7, SUSD2 down-regulation significantly increased invasion of SMMC7721 cells *(P* < 0.05).

## Discussion

As is apparent with most disease processes, HCC is more effectively treated when it is diagnosed at an early stage. Unfortunately, the main serum tumor markers including alpha-fetoprotein (AFP), lens culinaris agglutinin-reactive AFP (AFP-L3), des-gamma carboxyprothrombin (DCP) and glypican-3 (GPC3) [16], failed to reach the optimal results when tested in the surveillance and early detection [17]. Despite these advances in molecular diagnosis of HCC, Nam SW and his colleagues proposed a 3-gene signature, including GPC3, LYVE1 and survivin as an accurate molecular tool (>80 % accuracy) to discriminate dysplastic nodules and small HCC <2 cm in size, but restricted to HCV-related HCC [18]. Taken together, there is still an urgent need for us to identify novel biomarkers which have important functions during HCC progression and that may help us find better diagnostic markers or therapeutic targets.

Although recent studies have focused on the relationship between SUSD2 expression and multiple types of human cancer, such as breast, colon [13, 14], the expression pattern of SUSD2 protein and its biologic function has not been revealed in HCC. In the present study, we reported for the first time the SUSD2 expression levels of both the mRNA and protein were markedly reduced in the majority of HCC tissues, when compared with their paired adjacent normal liver tissues. Next, the expression of the SUSD2 protein was examined by IHC, using a TMA containing 180 HCC samples and 16 non-malignant liver tissues. IHC results demonstrated that SUSD2 high expression was observed in 100 % of non-malignant liver tissues when compared to that in the 37.8 % HCC tissues, suggesting that down-regulated of SUSD2 may provide a selective advantage in the HCC tumorigenic development and progression. Furthermore, in order to evaluate the association of SUSD2 expression with HCC patients’ clinicopathologic features, ROC analysis was carried out for each of the clinicopathological parameters, including histological grade, clinical stage, pT stage, pN stage, pM stage. The analysis results showed significant positive correlation between HCC and clinicopathological parameters we focused on, such as histological grade (χ^2^ = 5.198, *P* = 0.007), clinical stage (χ^2^ = 30.244, *P* = 0.000), pT status (χ^2^ = 33.175, *P* = 0.000) and pN status (χ^2^ = 4.785, *P* = 0.000), pM status (χ^2^ = 4.620,* P* = 0.032). These data provided evidence that the decreased expression of SUSD2 may play an important role in tumorigenic process of HCC and may serve as a potential marker for the diagnosis of HCC.

To investigate the possibility that SUSD2 could suppress the tumorigenesis of HCC, we altered the expression of SUSD2 in HCC cell lines by up-regulation and down-regulation. As expected, up-regulation of SUSD2 in HepG2 cells significantly inhibited the cell proliferation and promoted cell apoptosis compared with those of the cells transfected with empty vectors. Consistently, down-regulation of SUSD2 in SMMC7721 cells enhanced the cell proliferation and decreased cell apoptosis compared with those of the cells transfected with empty vectors. Taken together, these results suggested that SUSD2 played as a tumor suppressor and thus inhibited the growth of HCC tumors in vitro. Furthermore,in our study, we proved that up-regulation of SUSD2 significantly decreased the migration and ability of HepG2 cells to invade through Matrigel, a basement membrane that used to imitate the metastatic potential of cancer cells. Metastasis is the main cause of cancer recurrence and tumor-related death [19, 20], the absence of SUSD2 in HCC may represent advanced disease and poor prognosis for the patient.

The major finding from this study provide evidence that SUSD2 expressed in HCC were less than that in the adjacent liver normal tissues. A low level of SUSD2 is associated with clinicopathologic parameters and represents a more aggressive status of HCC. We have proven that up-regulation of SUSD2 may reverse tumor formation, making it a potentially effective biomaker,but the mechanisms of SUSD2 in HCC carcinogenesis and the prognostic value of SUSD2 in HCC patients are needed in further studies.

## Conclusion

In conclusion, SUSD2 was down-regulated in HCC tissues and related to histological grade, clinical stage, TNM stage. We also provided evidence demonstrating SUSD2 affected HCC cells proliferation, invasion, migration, apoptosis. SUSD2 may be a potential marker for the diagnosis of HCC.

## Abbreviations

SUSD2: Sushi Domain Containing 2
HCC: hepatocellular carcinoma
qRT-PCR: quantitative real-time polymerase chain reaction
WB: western blotting
IHC: immunohistochemistry
ROC: receiver operating characteristic
MA: tissue microarray
cDNA: complementary DNA
RIPA: radio-immunoprecipitation assay
PVDF: polyvinylidene fluoride
AUC: area under the ROC curve
